# Impact of Antidepressant Treatment on Fibronectin Levels in Patients with Depression and Chronic Heart Failure

**DOI:** 10.1192/j.eurpsy.2024.238

**Published:** 2024-08-27

**Authors:** A. K. Sikora, S. Fedorov

**Affiliations:** ^1^Psychiatry, Narcology and Medical Psychology; ^2^Ivano-FrankIskenderun National Medical University, Ivano-Frankivsk , Ukraine

## Abstract

**Introduction:**

Inflammation has emerged as a critical factor in the pathophysiology of both depression and chronic heart failure (HF). Chronic heart failure, a complex clinical syndrome, is often accompanied by a state of heightened inflammation, with elevated levels of proinflammatory markers. Likewise, depression, a prevalent comorbidity in HF patients, has been intricately linked to inflammation, with evidence suggesting a bidirectional relationship.

**Objectives:**

This study aimed to evaluate the effect of antidepressant treatment on plasma fibronectin levels in patients with comorbid depression and chronic heart failure.

**Methods:**

We enrolled a total of 113 patients with HF, all of whom had comorbid depression. The patients were divided into two groups based on the antidepressant treatment they received: Group 1 (n = 78) received vortioxetine, and Group 2 (n = 35) received sertraline. Before initiating treatment and after 6 months, we measured fibronectin levels in the patients’ plasma.

**Results:**

The study revealed a significant difference in the effects of the two antidepressants on fibronectin levels. Patients treated with vortioxetine demonstrated a substantial reduction in fibronectin levels post-treatment, with an approximate threefold decrease compared to the pre-treatment levels (pre-treatment value ± standard deviation) μg/ml to (post-treatment value ± standard deviation) μg/ml, (p<0.05). Conversely, patients treated with sertraline experienced a comparatively lesser reduction in fibronectin levels, with a change from (pre-treatment value ± standard deviation) μg/ml to (post-treatment value ± standard deviation) μg/ml (p<0.05).

**Conclusions:**

This study highlights the considerable impact of vortioxetine on fibronectin levels in patients with comorbid depression and chronic heart failure, resulting in a significant reduction. In contrast, sertraline’s effect on fibronectin levels, while present, is notably less pronounced. The study emphasizes the potential therapeutic benefit of vortioxetine in cardiac remodeling associated with depression in patients with chronic heart failure, underscoring the need for further research and exploration.

**Disclosure of Interest:**

None Declared

